# LINC00324 in cancer: Regulatory and therapeutic implications

**DOI:** 10.3389/fonc.2022.1039366

**Published:** 2022-12-22

**Authors:** Qing Xia, Jinze Shen, Qurui Wang, Yufei Ke, Qibin Yan, Hanbing Li, Dayong Zhang, Shiwei Duan

**Affiliations:** ^1^ Department of Clinical Medicine, Zhejiang University City College School of Medicine, Hangzhou, Zhejiang, China; ^2^ College of Pharmacy, Zhejiang University of Technology, Hangzhou, Zhejiang, China; ^3^ Key Laboratory of Novel Targets and Drug Study for Neural Repair of Zhejiang Province, School of Medicine, Zhejiang University City College, Hangzhou, Zhejiang, China

**Keywords:** LINC00324, cancer, RNA-Binding Protein, ceRNA, signaling pathway, prognosis

## Abstract

LINC00324 is a 2082 bp intergenic noncoding RNA. Aberrant expression of LINC00324 was associated with the risk of 11 tumors and was closely associated with clinicopathological features and prognostic levels of 7 tumors. LINC00324 can sponge multiple miRNAs to form complex ceRNA networks, and can also recruit transcription factors and bind RNA-binding protein HuR, thereby regulating the expression of a number of downstream protein-coding genes. LINC00324 is involved in 4 signaling pathways, including the PI3K/AKT signaling pathway, cell cycle regulatory pathway, Notch signaling pathway, and Jak/STAT3 signaling pathway. High expression of LINC00324 was associated with larger tumors, a higher degree of metastasis, a higher TNM stage and clinical stage, and shorter OS. Currently, four downstream genes in the LINC00324 network have targeted drugs. In this review, we summarize the molecular mechanisms and clinical value of LINC00324 in tumors and discuss future directions and challenges for LINC00324 research.

## Introduction

Next-generation sequencing technology has shown that most of the human genome can be transcribed into non-coding RNA (ncRNA), including microRNA (miRNA), circular RNA (circRNA), long non-coding RNA (lncRNA), and pseudogenes (1). ncRNAs play regulatory roles at gene transcription and post-transcription levels, and their abnormal expression is often associated with the pathogenesis of various diseases (2, 3). Among them, lncRNAs have been shown to regulate various gene expression processes. By recruiting transcription factors to interact with transcription initiation sites, lncRNAs can act as transcriptional co-activators, or as scaffolds for proteins (4). Similarly, lncRNAs can also act as molecular decoys to capture transcription factors, thereby limiting their ability to bind to DNA binding sites (5). In addition to transcriptional regulation, lncRNAs also participate in mRNA splicing, act as miRNA sponges or compete for miRNA binding sites on mRNAs, and play important roles in mRNA processing, maturation, and stability (6). In recent years, abnormal expression and mutation of lncRNAs are closely related to the differentiation, proliferation, metastasis, and apoptosis of cancer cells. Current studies have shown that LncRNAs can be used as biomarkers for cancer diagnosis and prognosis (7, 8).

LINC00324, also known as c17orf44, is located on human chromosome 17p13.1 and is a 2082 bp long insertion/intergenic noncoding RNA (9). LINC00324 was first found to be significantly differentially expressed between tamoxifen-treated and non-treated breast cancer subtypes (10). LINC00324 is aberrantly expressed in 11 cancers, and dysregulated LINC00324 is also associated with poor prognosis and clinical characteristics, including tumor size, lymph node metastasis, tumor TNM stage, and clinical stage in cancer patients. LINC00324 is involved in multiple oncogenic molecular pathways that can affect cell proliferation, migration, invasion, and apoptosis. In this review, we summarize the mechanism of action and clinicopathological features of LINC00324 in tumors and discuss future directions and challenges for LINC00324 research.

## Abnormal expression of LINC00324 in tumors

Aberrant expression of LINC00324 was significantly associated with oncogenic risk in 11 cancers. These cancers involve the human respiratory, digestive, nervous, endocrine, and reproductive systems. LINC00324 is upregulated in cancer tissues and cell lines in the respiratory system, including NSCLC (11), NPC (12), and LAC (13). In the digestive system, LINC00324 was highly expressed in tumor tissues and cell lines of GC (14, 15), SaOS (16), and HCC (17). In addition, a higher expression of LINC00324 was also found in the cell line of CRC (18). In nervous system tumors, LINC00324 is aberrantly overexpressed in RB (19). In the endocrine system, LINC00324 was upregulated in tumor tissues and cell lines of PTC (15, 20). In addition, the expression level of LINC00324 was also higher in the plasma of IDD patients (21). In the reproductive system, abnormally high expression of LINC00324 was also present in tumor tissues and cell lines of IOT (9). In contrast, LINC00324 was lowly expressed in BC cell lines and tissues (22).

## Molecular mechanisms by which LINC00324 affects tumor development

**Figure 1:**
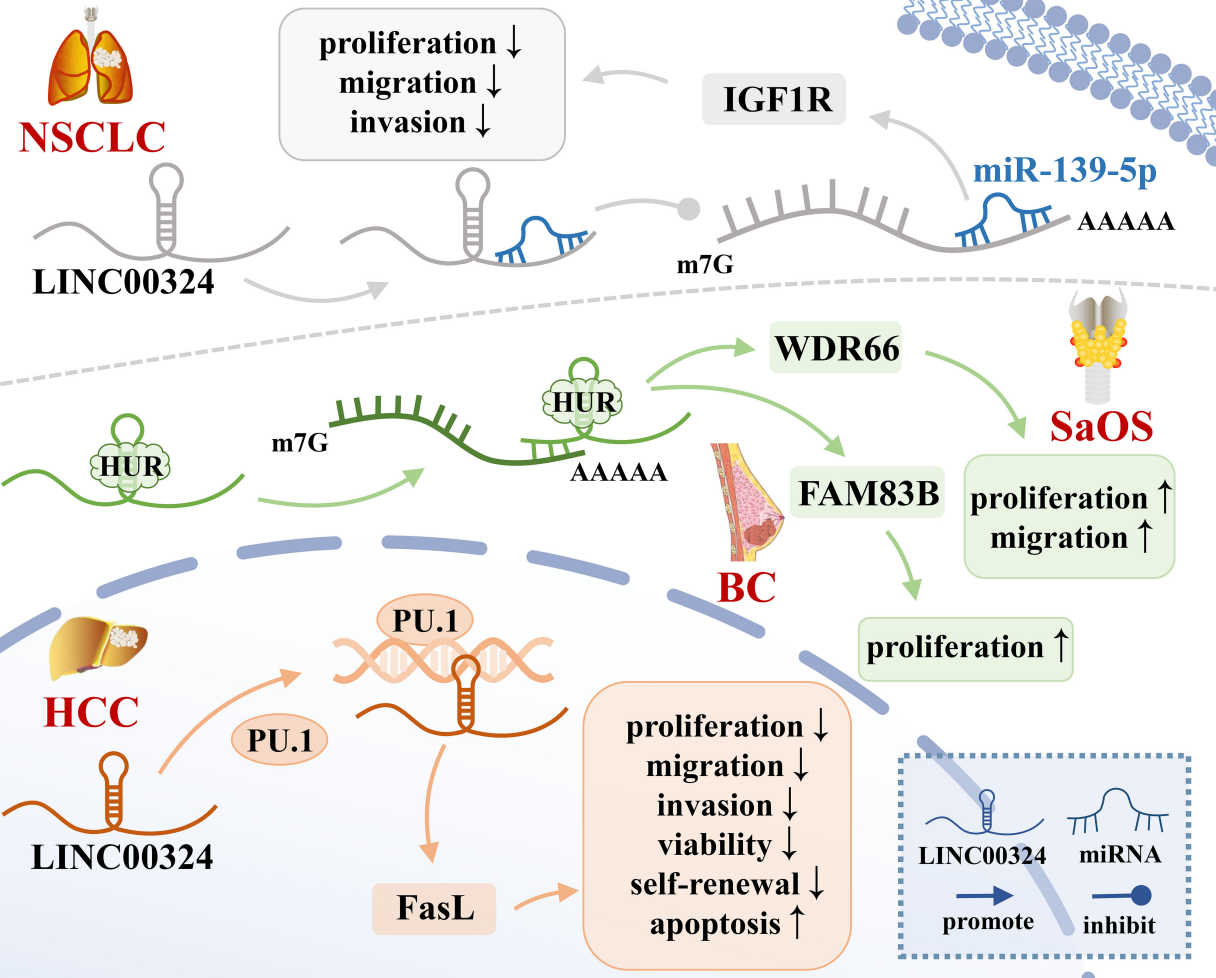
The mechanisms of LINC00324 in cancer. BC, Breast cancer; HCC, Hepatocellular carcinoma; NSCLC, Non-small cell lung carcinoma; SaOS, Osteosarcoma.

**Figure 2:**
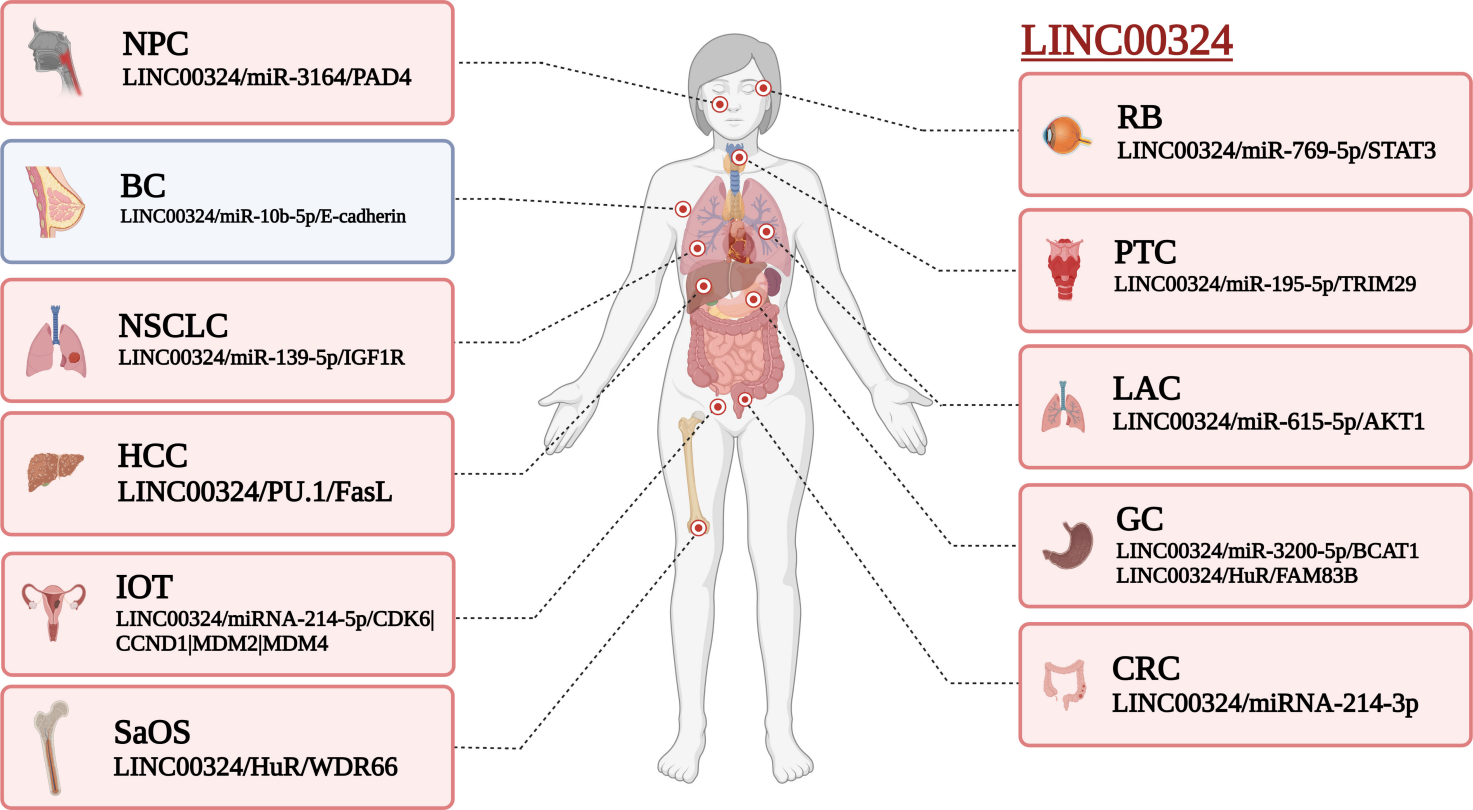
Effect of LINC00324 on cancer development. Red represents the mechanism of promoting cancer, and blue represents the mechanism of inhibiting cancer. BC, Breast cancer; CRC, Colorectal cancer; GC, Gastric cancer; HCC, Hepatocellular carcinoma; IOT, Immature ovarian teratocarcinoma; LAC, Lung adenocarcinoma; NPC, Nasopharyngeal carcinoma; NSCLC, Non-small cell lung carcinoma; PTC, Papillary thyroid cancer; RB, Retinoblastoma; SaOS, Osteosarcoma. (created by BioRender).

As shown in **Table 1**, LINC00324 is involved in multiple molecular mechanisms to regulate tumor development. Specifically, LINC00324 can bind RNA-binding protein (RBP) to enhance messenger RNA (mRNA) stability, act as a molecular sponge for miRNAs to regulate downstream mRNA expression, and even recruit transcription factors to promote downstream gene transcription (**Figures 1**, **2**).

**Table 1:** Regulatory mechanisms of LINC00324 in cancer.

Diseases	Assessed cell lines	LINC00324 expression	Effects *in vivo*	Effects of LINC00324 knockdown *in vitro*	Regulatory mechanism	Reference
BC	MDA-MB-231, MCF-7 and MCF-10A	down-regulated	Overexpression of LINC00324 reduces tumor volume	cell cycle↑, proliferation↑, colony formation↑, invasion↑, migration↑, and apoptosis↓	LINC00324/miR-10b-5p/E-cadherin	(22)
CRC	SW620, HCT15, SW480 and HCT116	upregulated	—	proliferation↓, invasion↓, and migration↓	LINC00324/miRNA-214-3p; cell cycle regulatory pathway	(18)
GC	AGS, MGC803, MKN-45, and GES-1	upregulated	—	proliferation↓, invasion↓, and migration↓	LINC00324/miR-3200-5p/BCAT1	(14)
	SGC7901 and BGC823	upregulated	Knockdown of LINC00324 reduces tumor volume	proliferation↓, invasion↓, migration↓, and apoptosis↑	LINC00324/HuR/FAM83B	(15)
HCC	HuH-7 and THLE-3	upregulated	Knockdown of LINC00324 reduces tumor volume	proliferation↓, invasion↓, migration↓, apoptosis↑, viability↓, and self-renewal↓	LINC00324/PU.1/FasL	(17)
IOT	Hs38.Tand PA-1	upregulated	—	viability↓, colony formation↓, and apoptosis↑	LINC00324/miRNA-214-5p/CDK6, CCND1, MDM2, MDM4,;cell cell cycle regulatory pathway	(9)
LAC	A549, PC-9, H1650, SPCA1, and H1299	upregulated	—	proliferation↓, migration↓, invasion↓, and apoptosis↑	LINC00324/miR-615-5p/AKT1	(13)
	16HBE					
NPC	5-8F, 6-10B, and NP69	upregulated	Knockdown of LINC00324 reduces tumor volume	proliferation↓, autophagy↓, apoptosis↑, and viability↓	LINC00324/miR-3164/PAD4;PI3K/AKT pathway	(12)
NSCLC	A549, H1299, H460, SK-MES-1, and SPC-A-1	upregulated	—	proliferation↓, invasion↓, and migration↓	LINC00324/miR-139-5p/IGF1R	(11)
PTC	Nthy-ori 3-1,K1,TPC-1,BCPAP,KTC-1	upregulated	—	cell cycle↓, proliferation↓, and apoptosis↑	Notch signaling pathway	(20)
	B-CPAP, KTC-1, TPC1, K1, Nthy-Ori 3-1, and 293T	upregulated	—	proliferation↓ and invasion↓	LINC00324/miR-195-5p/TRIM29	(36)
RB	Y79 and WERI-RB-1	upregulated	Knockdown of LINC00324 reduces tumor volume	cell cycle↓, proliferation↓, colony formation↓, invasion↓, migration↓, and apoptosis↑	LINC00324/miR-769-5p/STAT3; Jak/STAT3 signaling pathway	(19)
SaOS	hFOB1.19, 143B, MG-63, Saos-2 and HOS	upregulated	—	proliferation↓ and migration↓	LINC00324/HuR/WDR66	(16)

BC, Breast cancer; CRC, Colorectal cancer; GC, Gastric cancer; HCC, Hepatocellular carcinoma; IOT, Immature ovarian teratocarcinoma; LAC, Lung adenocarcinoma; NPC, Nasopharyngeal carcinoma; NSCLC, Non-small cell lung carcinoma; PTC, Papillary thyroid cancer; RB, Retinoblastoma; SaOS, Osteosarcoma;"↑" means increase and "↓" means decrease.

### Interaction of LINC00324 with RBP

RBP is an important class of proteins in cells. Their special RNA-binding domains can interact with RNA and are widely involved in multiple post-transcriptional regulatory processes of RNA, including splicing, transport, sequence editing, intracellular localization, and translation control (24). HuR is a common RBP that binds adenylate/uridylate-rich elements (AREs) on the 3’-untranslated region (3’-UTR) of gene transcripts, thereby enhancing the mRNA stability (15). LINC00324 recruits HuR to enhance mRNA stability of WDR66 and FAM83B (15, 16), thereby promoting tumor development.

WDR66 is a member of a family of proteins containing WD repeats that promote the occurrence and progression of ESCC and GC (25). In SaOS, LINC00324 binds to HuR, thereby increasing the stability of WDR66 mRNA, ultimately promoting the proliferation and migration of SaOS cells (16). FAM83B is an oncogene that is closely related to EGFR/RAS/MAPK signaling and is able to activate the PI3K/AKT/mTOR signaling pathway in breast cancer, thereby promoting cell proliferation, anchoring independent growth (AIG), and tumorigenesis (26). In GC, LINC00324 can enhance the stability of FAM83B mRNA by binding to HuR, thereby promoting gastric cancer cell proliferation (15).

### The ceRNA network of LINC00324 in cancer

**Figure 3:**
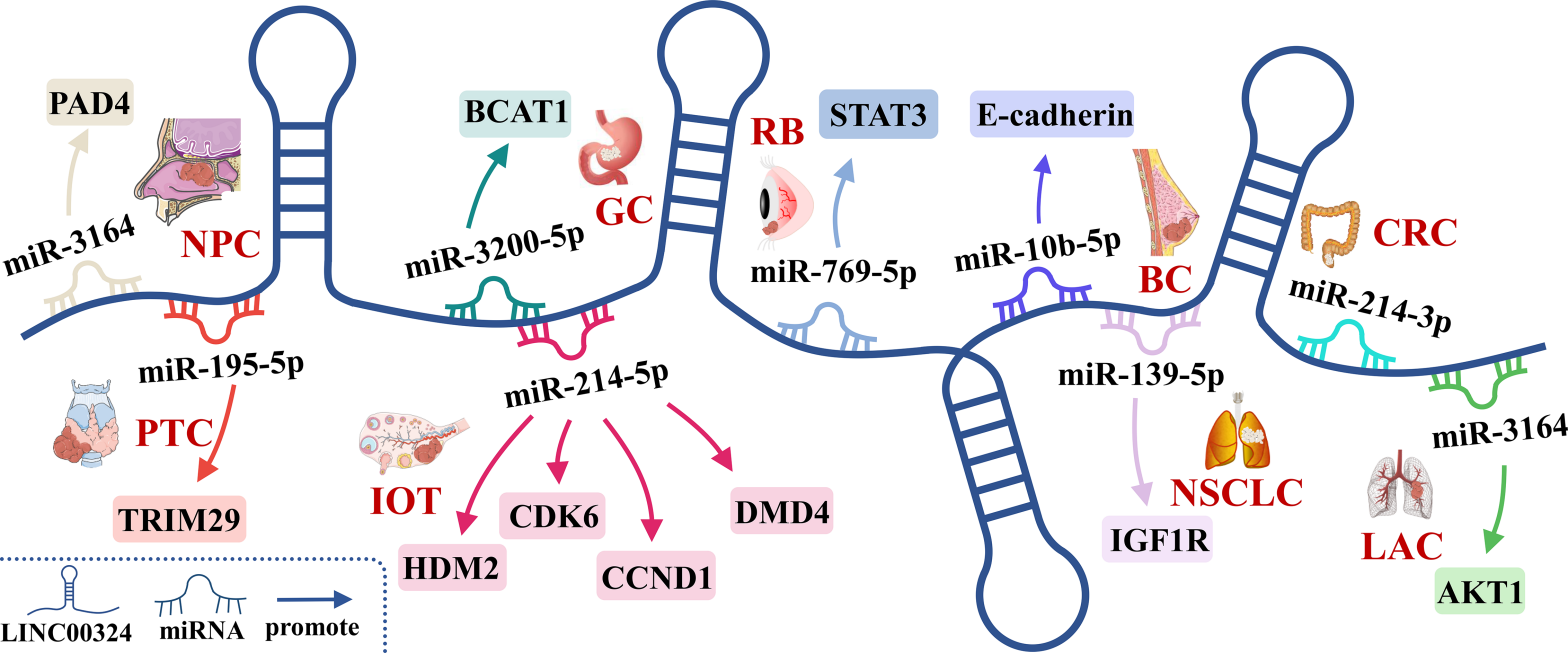
The competing endogenous RNA (ceRNA) mechanism and potential downstream regulatory mechanisms of LINC00324. BC, Breast cancer; CRC, Colorectal cancer; GC, Gastric cancer; IOT, Immature ovarian teratocarcinoma; LAC, Lung adenocarcinoma; NPC, Nasopharyngeal carcinoma; NSCLC, Non-small cell lung carcinoma; PTC, Papillary thyroid cancer; RB, Retinoblastoma.

The lncRNAs are often stably expressed in the cytoplasm, which contributes to the efficient binding of miRNAs and coexists with miRNAs in the RNA-induced silencing complex (RISC) (27). As shown in **Table 2** and **Figure 3**, LINC00324 exerts tumor-promoting effects through 7 ceRNA axes. These include LINC00324/miR-139-5p/IGF1R axis in NSCLC, LINC00324/miR-214-5p/CDK6|CCND1|MDM2|MDM4 axis in IOT, LINC00324/miR-3164/PAD4 axis in NPC, LINC00324/miR-769-5p/STAT3 axis in RB, LINC00324/miR-3200-5p/BCAT1 axis in GC, LINC00324/miR-615-5p/AKT1 axis in LAC, LINC00324/miR-195 -5p/TRIM2 axis in PTC, and LINC00324/miR-10b-5p/E-cadherin axis in breast cancer.

**Table 2:** MiRNAs regulated by LINC00324 and corresponding binding sites.

Cancers	miRNAs	Binding site of LINC00324	Binding site of miRNAs	Reference
BC	miR-10b-5p	UUuuuUUuUaagACAGGGU	AAgccAAgAUGUCCCA	(22)
CRC	miR-214-3p	CCUGCUG	GGACGAC	(18)
GC	miR-3200-5p	GUGuaaagUCUCAGAU	CACgcggaAGAGUGUA	(14)
IOT	miR-214-5p	GACAGGC	CUGUCCG	(9)
LAC	miR-615-5p	GCCCCUG	CGGGGGC	(13)
NPC	miR-3164	CCCaUcAAGUCAC	GGGaAuUUCAGUG	(12)
NSCLC	miR-139-5p	CUGUAGG	GACAUCU	(11)
PTC	miR-195-5p	UUaUUGCUGCU	AAgAcACGACGA	(36)
RB	miR-769-5p	AGGUCUC	UCCAGAG	(19)

BC, Breast cancer; CRC, Colorectal cancer; GC, Gastric cancer; IOT, Immature ovarian teratocarcinoma; LAC, Lung adenocarcinoma; NPC, Nasopharyngeal carcinoma; NSCLC, Non-small cell lung carcinoma; PTC, Papillary thyroid cancer; RB, Retinoblastoma.

As a receptor for IGF-1 and IGF-2, IGF1R plays an important role in promoting cell proliferation and differentiation and inhibiting apoptosis (28). In NSCLC, the LINC00324/miR-139-5p/IGF1R axis promotes cell proliferation, differentiation, and invasion and inhibits apoptosis (11). Cyclin-dependent kinase 6 (CDK6) is a cell cycle-dependent kinase that induces angiogenesis, inhibits the cell cycle, activates stem cells, and promotes gene transcription of immune responses (29). CyclinD1 is an important sensor and activator of cell cycle initiation and progression. The complex formed by the CyclinD1 protein encoded by CCND1 and CDK6 can regulate cell cycle G1 phase progression and G1/S transition (30). MDM2 and MDM4 are negative regulators of the tumor suppressor gene TP53, which can inhibit the transcriptional activity of p53 and promote the degradation of p53 by the proteasome, thereby promoting the occurrence and development of tumors (31). In IOT, the LINC00324/miR-214-5p/CDK6|CCND1|MDM2|MDM4 axis promotes cancer cell proliferation and inhibits cancer cell apoptosis (9). PAD4 is an oncogene that promotes CRC liver metastases by driving extracellular matrix citrullination (32). In NPC, the LINC00324/miR-3164/PAD4 axis promotes NPC cell proliferation and inhibits apoptosis and autophagy (12). STAT3 is a key transcription factor belonging to the STAT family that promotes cancer cell proliferation, and invasion, and also induces angiogenesis and immunosuppression (33). In RB, the LINC00324/miR-769-5p/STAT3 axis increases cell proliferation, colony formation, migration, invasion, and tumorigenesis, and inhibits apoptosis (19). Branched-chain aminotransferase 1 (BCAT1) is involved in the metabolism of branched-chain amino acids, provides an energy source for tumor growth, and participates in various biosynthetic pathways of tumors (reviewed). In GC, the LINC00324/miR-3200-5p/BCAT1 axis promotes malignancy progression (14). Akt is a serine/threonine kinase encoded by AKT1 that is involved in the regulation of cell survival, proliferation, migration, metabolism, and angiogenesis (34). In LAC, the LINC00324/miR-615-5p/AKT1 axis promotes LAC cell proliferation and development (35). TRIM29 is a tumorigenic factor that negatively regulates p53 to induce malignant transformation of cells, increase radioresistance, and generate anti-apoptotic responses (36). In PTC, the LINC00324/miR-195-5p/TRIM2 axis enhances cell proliferation and invasion (23).

In contrast to the above, LINC00324 is lowly expressed in BC. E-cadherin is mainly used to maintain tight junctions between cells (37), and deletion of this protein promotes epithelial-mesenchymal transition (EMT) in cancer cells (38). EMT can lead to weakened connections between cancer cells, increased proliferation and invasion, and a high frequency of distant metastasis (39). In BC, the low expression of LINC00324 can lead to the up-regulation of miR-10b-5p expression, which in turn inhibits the expression of the downstream target gene E-cadherin, thereby promoting cell proliferation, invasion, and migration, while inhibiting apoptosis (22).

In conclusion, in BC, IOT, LAC, NPC, NSCLC, PTC, and RB, LINC00324 regulates the ceRNA axes to promote tumor cell proliferation, migration, and invasion.

### Recruitment of transcription factors by LINC00324 in cancer

Highly expressed LINC00324 promotes cancer development by recruiting transcription factors to increase downstream gene transcription. PU.1 is an important transcription factor that opens chromatin and binds to downstream gene promoters to promote the transcription of downstream effector genes (40). Fas ligand (FasL), a member of the TNF-α family, is often highly expressed in HCC cells and can induce apoptosis of peripheral cells through autocrine or paracrine FasL (41). Resistance to Fas/FasL-induced apoptosis is related to the degree of tumor malignancy (42). In HCC, LINC00324 can promote the transcription of FasL by recruiting PU.1 to the FasL promoter region, up-regulating the level of FasL in liver cancer stem cells, inhibiting cell apoptosis, and promoting the occurrence and development of cancer (17).

## LINC00324-related signaling pathways in cancer

**Figure 4:**
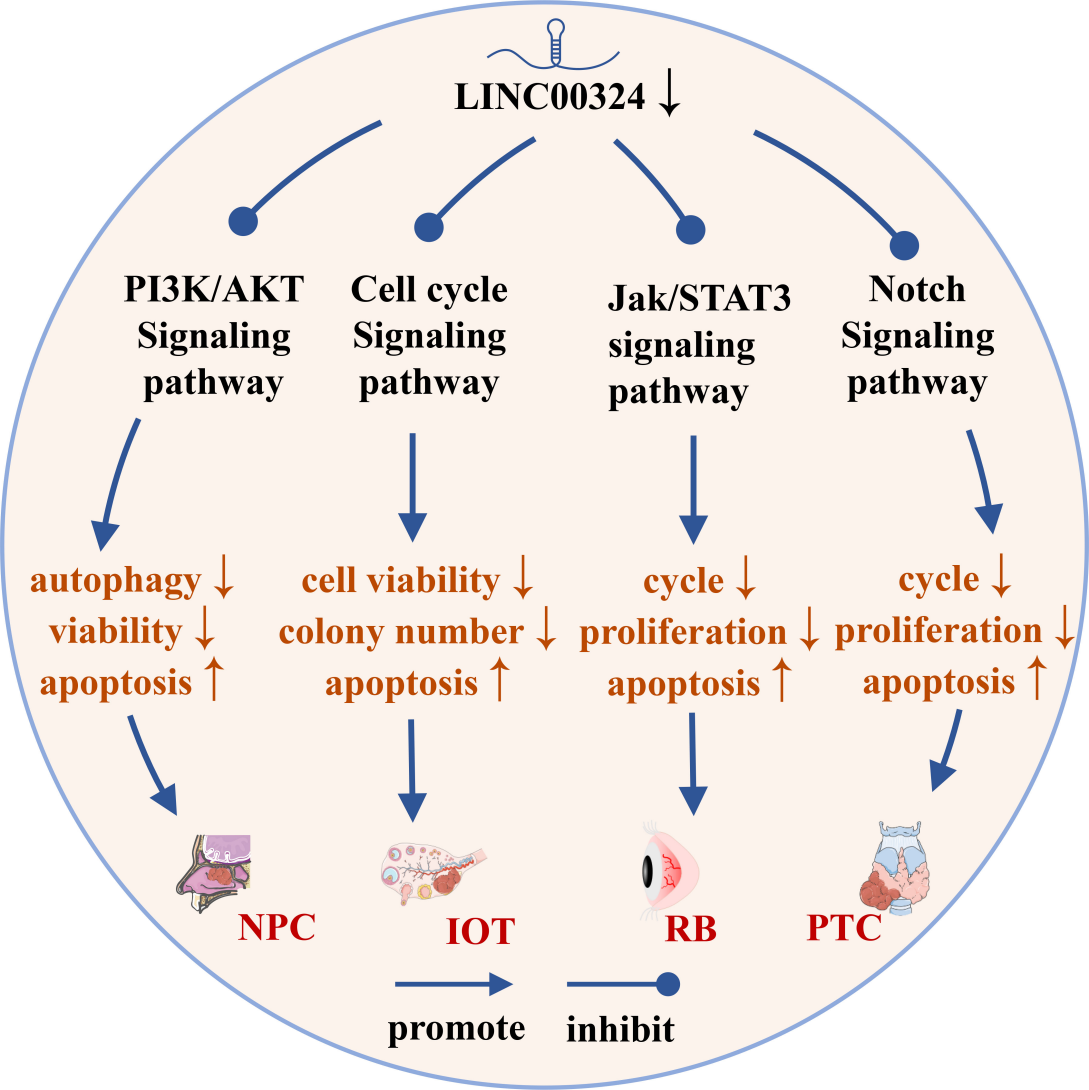
The role of LINC00324 in signaling pathways. IOT, Immature ovarian teratocarcinoma; NPC, Nasopharyngeal carcinoma; PTC, Papillary thyroid cancer; RB, Retinoblastoma.

As shown in **Figure 4**, LINC00324 can participate in the regulation of four signaling pathways in cancer, including the PI3K/AKT signaling pathway, p53 signaling pathway, cell cycle regulatory pathway, and Notch signaling pathway.

### LINC00324 and the PI3K/AKT signaling pathway

The PI3K/AKT pathway is an important signaling pathway involved in the normal cellular process, but its abnormal activation inhibits autophagy and apoptosis of various cancer cells (43). LINC00324 modulates the PI3K/AKT signaling pathway and promotes cancer development in NPC, NSCLC, and LAC.

Peptidyl arginine deiminase 4 (PAD4) is an enzyme that converts arginine to citrulline, and upregulation of PAD4 activates the PI3K/AKT pathway to promote NPC malignancy (44). In NPC, LINC00324 activates the PI3K/AKT pathway to inhibit apoptosis and autophagy by interacting with miR-3164 and recruiting HuR protein to upregulate PAD4 expression, thereby promoting NPC malignancy (12).

Insulin-like growth factor 1 (IGF1) and IGF1 receptor (IGF1R) belong to the insulin-like growth factor family. IGF1 specifically binds to the membrane receptor IGF1R and activates the intracellular insulin receptor substrate (IRS), which in turn activates the PI3K/AKT pathway and promotes cancer development (45). In NSCLC, highly expressed LINC00324 can sponge miR-139-5p and upregulate IGF1R expression, thereby activating the PI3K/AKT pathway to promote NSCLC cell proliferation, differentiation, and invasion, and inhibit apoptosis (28). In LAC, the highly expressed LINC00324 can competitively inhibit miR-615-5p and upregulate the expression of Akt, thereby activating the PI3K/AKT pathway to promote the proliferation and development of LAC cells (35).

### LINC00324 and the cell cycle regulatory pathway

The cell cycle regulatory pathway regulates the cell cycle by regulating cyclin-dependent kinase (CDK) and cyclin and is closely related to the occurrence, development, and metastasis of tumors (46). Cyclin has a promoting effect on CDK. The CDK/cyclin complex formed by cyclin and CDK can phosphorylate Rb, causing Rb to release E2F originally bound to it, and at the same time activate the transcription of E2F-dependent transcription factors to drive cell cycle progression (47). Abnormal expression of CDK4/6 in various cancers can lead to dysregulation of the G1-S checkpoint of the cell cycle to promote cancer cell proliferation (48).

The tumor suppressor gene TP53 is mutated in 50% of tumors. p53 is a key factor regulating stem cell differentiation, which can inhibit the induction of differentiated cells into pluripotent stem cells and the generation of cancer stem cells (49). p21, also known as Cyclin-dependent kinase inhibitor 1A, is a CDK inhibitor that plays an important role in controlling cell cycle progression (50). p53 activation induces p21 expression to inactivate CDKs, which in turn arrests the G1 and G2 phases of the cell cycle, thereby inhibiting the cell cycle progression (51). LINC00324 regulates the cell cycle pathway in IOT and CRC to promote cancer development.

In the IOT, the target genes of miR-214-5p were mainly enriched in the p53 signaling pathway. High expression of LINC00324 can sponge miR-214-5p, thereby upregulating MDM2 and MDM4 expression, and inhibiting p53 activity to reduce p21 expression (52). Meanwhile, miR-214-5p can also downregulate CyclinD and CDK4/6 levels to promote IOT cell proliferation and inhibit apoptosis (9). In CRC, the LINC00324/miR-214-3p axis, downregulate p21, upregulates Cyclin D, MMP-2, MMP-9, and MMP-14 levels, thereby promoting CRC cell proliferation, migration, and invasion (18).

### LINC00324 and the Notch signaling pathway

The Notch signaling pathway is involved in important processes such as hematopoiesis and cardiac development, and its abnormal activities are closely related to tumors, autoimmune diseases and congenital diseases (53). Notch signaling pathway can be mainly mediated by paracrine to promote the formation of tumor microenvironment and participate in the development of cancer (54). In PTC, LINC00324 promotes PTC cell proliferation and blocks apoptosis by inhibiting Notch signaling pathway (20).

### LINC00324 and the Jak/STAT3 signaling pathway

Abnormal activation of the Jak/STAT3 signaling pathway increases the probability of stem cell transition and eventually induces cancer (55). When the cell membrane receptor is stimulated by extracellular signals, it activates the non-receptor tyrosine kinase Jak, which phosphorylates a conserved tyrosine residue on STAT3 in the cytoplasm to form p-STAT3 (56). After dimerization, p-STAT3 enters the nucleus and binds to specific genes to exert transcriptional activity, thereby activating the Jak/STAT3 signaling pathway (57). In RB, high expression of LINC00324 competitively inhibits miR-769-5p, upregulates the expression of STAT3, and then activates the Jak/STAT3 signaling pathway to promote tumor progression (19).

## Clinical value of LINC00324 in cancer

As shown in **Table 3**, the abnormal expression of LINC00324 is closely related to the clinicopathological characteristics of various tumors and the prognosis of cancer patients. In RB, LINC00324 upregulation was associated with a more advanced tumor-node-metastasis (TNM) stage and a higher degree of optic nerve invasion (19). In NSCLC, LINC00324 upregulation was associated with a higher rate of lymph node metastasis (11). In GC, LINC00324 upregulation was significantly associated with a more advanced TNM stage, larger tumor, and a higher rate of lymph node metastasis (14, 15). In HCC and SaOS, LINC00324 upregulation was significantly associated with larger tumors, more advanced clinical stage, more malignant tumor differentiation, and more advanced lymph node metastasis (17) (16). Low expression of LINC00324 can also be used as a marker for better overall survival (OS) in SaOS patients (16). In BC, however, LINC00324 downregulation was significantly associated with larger tumors (22).

**Table 3:** Clinicopathological and prognostic values of LINC00324 in cancer.

Cancers	LINC00324 expression	Clinical samples	Clinicopathological characteristics	Prognosis	Reference
BC	deregulated	960 patients *via* TCGA and GEO database; 39 BC patients	Larger tumor size and volume	worse OS	(22)
GC	upregulated	66 paired GC tissues and adjacent normal tissues	more advanced TNM stage;larger tumor size; higher lymph node metastasis	worse OS and DFS	(15)
	upregulated	60 GC patients	larger tumor size; higher lymphoma metastasis;more advanced TNM stage	—	(14)
HCC	upregulated	125 patients *via* GEO database	larger tumor size; more advanced clinical stage;higher tumor differentiation degree; higher lymph node metastasis	worse OS	(17)
IOT	upregulated	45 paired IOT tissues and MOT tissues	—	worse OS	(9)
NSCLC	upregulated	48 NSCLC tissues and 18 adjacent normal tissues	higher rate of lymph node metastasis	worse OS	(11)
RB	upregulated	47 RB tissues and 13 normal retinal tissues	more advanced TNM stage;higher optic nerve invasion degree	worse OS	(19)
SaOS	upregulated	86 osteosarcoma tissues and 86 noncancerous tissues	larger tumor size; further distant metastasis; more advanced TNM stage; more malignant differentiated degree	worse OS	(16)

In RB, NSCLC, HCC, BC, IOT, and SaOS, high expression levels of LINC00324 are generally associated with poorer OS (9, 11, 16, 17, 19, 22). In GC, high expression levels of LINC00324 are often associated with poor OS, and disease-free survival (DFS) (14, 15). In BC, high expression of LINC00324 predicted better OS in BC patients (58).

**Figure 5:**
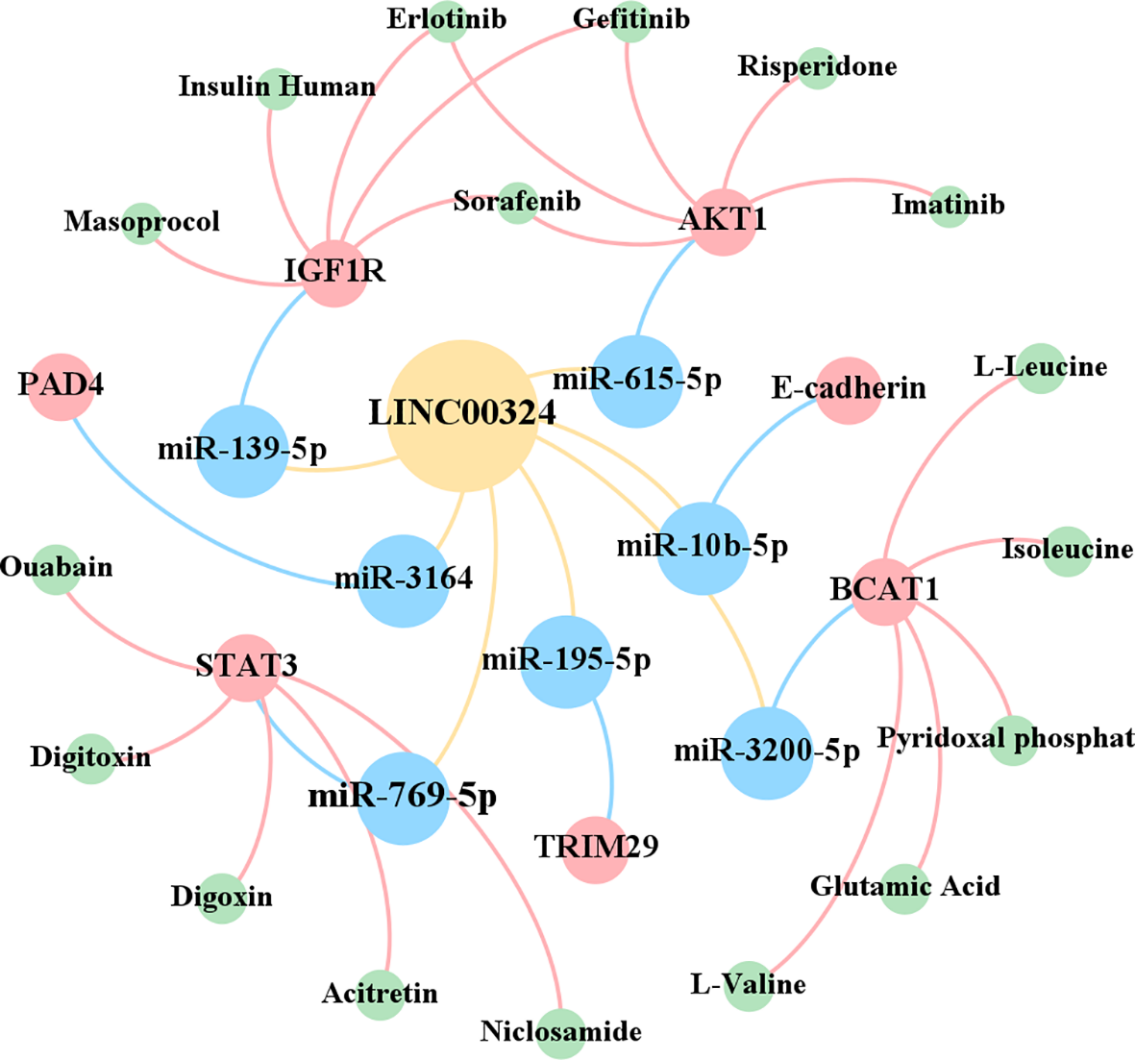
Therapeutic drugs related to target genes.

As shown in **Figure 5**, in the CADDIE database (59), we found that the downstream genes STAT3, AKT1, IGF1R, and BCAT1 of LINC00324 have multiple targeted drugs. They include STAT3-targeted drugs (Digoxin, Acitretin, Ouabain, Digitoxin, and Niclosamide), AKT1-targeted drugs (Gefitinib, Sorafenib, Erlotinib, Imatinib, and Risperidone), IGF1R-targeted drugs (Insulin human, Sorafenib, Masoprocol, Erlotinib, and Gefitinib), and BCAT1-targeted drugs (Pyridoxal phosphate, Glutamic Acid, L-Leucine, L-Valine, and Isoleucine). Future confirmation of the effects of LINC000324 would be interfered with by these drugs.

## Discussion

LINC00324 may have different regulatory mechanisms in different types of cancer. This may be related to tumor heterogeneity and limited sample size, as well as to different backgrounds of gene expression and different origins of tumor tissue. For example, the association of LINC00324 with RNA-modifying genes is also cancer-specific. In addition, the interaction between LINC00324 and the neighboring gene AURKB is complex and tumor-specific. The research of LINC00324 is still in the early stage. Diversified analytical methods can be introduced in future studies, which will help to more efficiently explore the potential mechanism of LINC00324 in cancer and accelerate the development of clinical applications.

Furthermore, the expression of LINC00324 in 10 cancer cells or tissues was generally higher than that in the corresponding normal cells or tissues. The bioinformatics analysis of TCGA also indicated that LINC00324 has a cancer-promoting effect in most cancers. Up-regulated LINC00324 was strongly associated with larger tumors, a higher degree of metastasis, later TNM staging, and poorer OS in multiple cancers. However, since a large body of current research has focused on the function of LINC00324 in promoting cancer development (60), the relationship between LINC00324 and clinical features has only been documented in a few cancer types. Many of the molecular mechanisms of LINC00324 have only been accomplished in the laboratory, but this still needs to be further explored in more cancers in the future.

Chemotherapy is a common means of treating cancer, but the metabolizing enzymes of chemotherapeutic drugs can mediate the inactivation of chemotherapeutic drugs and thus lead to drug resistance (61). The sensitivity of tumors to chemotherapeutic drugs often determines the efficacy of treatment. There is increasing evidence that lncRNAs can modulate chemoresistance by interacting with DNA, RNA, and proteins (62). At the same time, previous studies have confirmed that the downstream genes of LNC00324 can induce cancer cells to develop resistance to chemotherapy, including IGF-1R, AKT1, and STAT3. In NSCLC, IGF1R can cause the resistance of NCI-H1299 cells to EGFR kinase inhibitors (63). In ovarian cancer, AKT1 induces cisplatin resistance in OV2008 cells (64). In osteosarcoma, STAT3 induces resistance to doxorubicin and cisplatin in Saos-2 and U2-OS cells (65). However, there is still no research on the relationship between LINC00324 and tumor response to chemotherapy, so we believe that the relationship between LINC00324 expression and chemotherapy response can be the focus of research in the future.

## Conclusion

**Figure 6:**
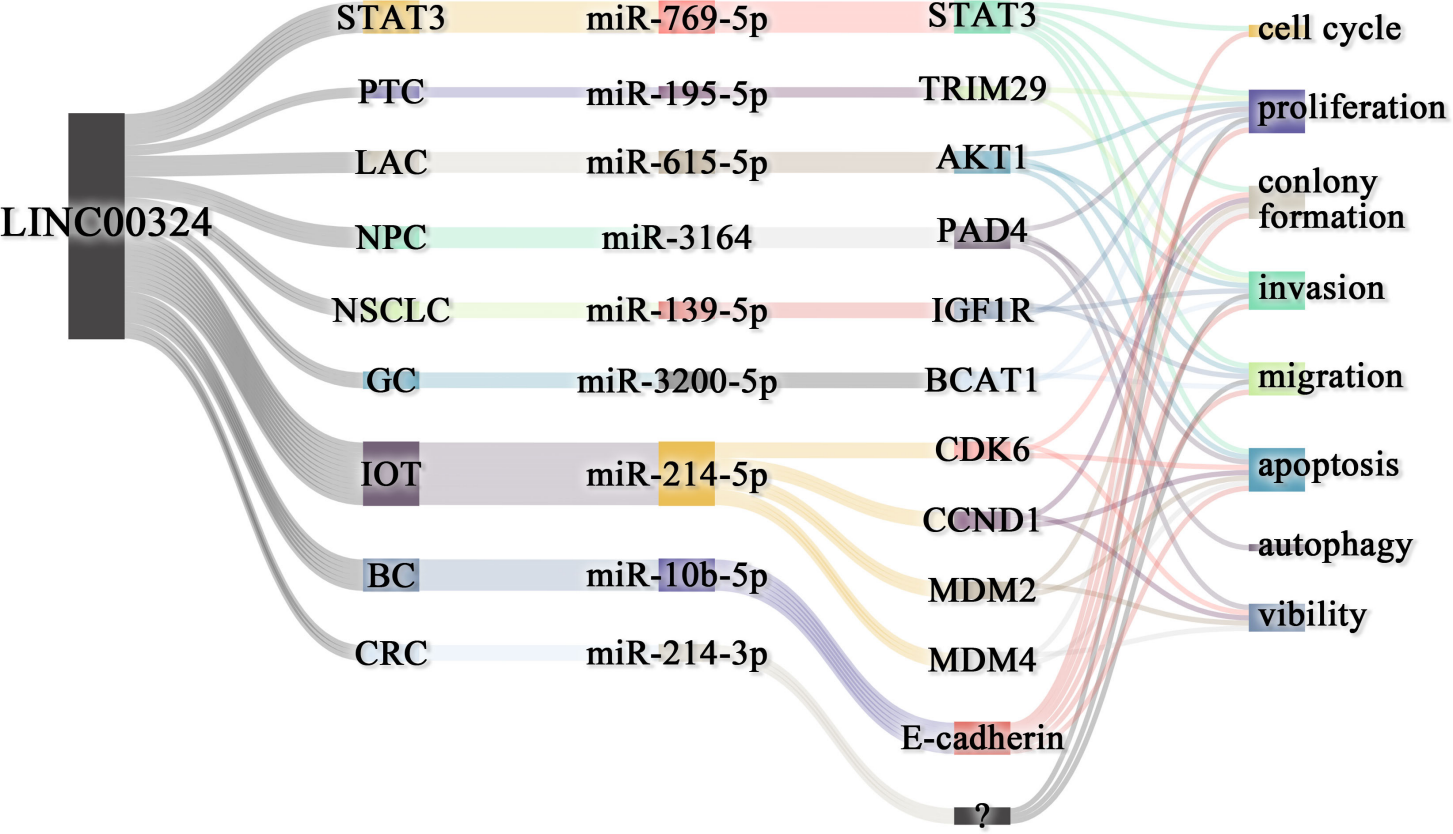
LINC00324 regulates cancer phenotype through ceRNA axes. BC, Breast cancer; CRC, Colorectal cancer; GC, Gastric cancer; LAC, Lung adenocarcinoma; NPC, Nasopharyngeal carcinoma; NSCLC, Non-small cell lung carcinoma; PTC, Papillary thyroid cancer; RB, Retinoblastoma.

Our work systematically elucidates the molecular mechanism of action of LINC00324 in tumors. INC00324 interacts with HuR and promotes tumor development by stabilizing the expression of WDR66 and FAM83B. LINC00324 acts as a miRNA sponge to affect the translation of nine downstream mRNAs, thereby promoting cancer phenotype and malignancy (**Figure 6**). LINC00324 can promote the transcription of the downstream effector gene FasL by recruiting the transcription factor PU.1 to promote cancer progression. In addition, LINC00324 can participate in multiple important signaling pathways and promote the development of cancer. In addition, We discuss the prognostic value and future research directions of LINC00324 in cancer development. In conclusion, dysregulated LINC00324 directly or indirectly has broad and complex effects on cancer development. An in-depth study of the role and mechanism of LNC00324 in cancer will help LINC00324 to become a therapeutic target and provide new applications for efficient and rapid diagnosis and individualized treatment of cancer.

## Author contributions

QX, JS, QW, YK, QY, and HL collected and analyzed the literature, drafted the figures and wrote the paper; SD and DZ conceived and gave the final approval of the submitted version. All authors contributed to the article and approved the submitted version.

## Glossary

**Table:**

3'-UTR	3' -untranslated region
AIG	Anchoring independent growth
AREs	Adenylate/uridylate-rich elements
BC	Breast cancer
ceRNA	competing endogenous RNA
circRNA	circular RNA
CRC	Colorectal cancer
DFS	Disease-free survival
EMT	Epithelial-mesenchymal transition
ESCC	Esophageal squamous cell carcinoma
FasL	Fas ligand
GC	Gastric cancer
HCC	Hepatocellular carcinoma
IDD	Intervertebral disk degeneration
IOT	Immature ovarian teratocarcinoma
LAC	Lung adenocarcinoma
lncRNA	Long non-coding RNA
miRNA	microRNA
MM	Multiple myeloma
mRNA	messenger RNA
ncRNA	non-coding RNA
NP	Nucleus pulposus
NPC	Nasopharyngeal carcinoma
NSCLC	Non-small cell lung carcinoma
OS	Overall survival
PTC	Papillary thyroid cancer
RB	Retinoblastoma
RBP	RNA-binding protein
RISC	RNA-induced silencing complex
SaOS	Osteosarcoma
TNM	Tumor-node-metastasis

## References

[1] Chan JJ Tay Y. Noncoding RNA:RNA regulatory networks in cancer. Int J Mol Sci (2018) 19(5):1310. doi: 10.3390/ijms19051310 29702599 PMC5983611

[2] Shen J Wu Y Ruan W Zhu F Duan S. miR-1908 dysregulation in human cancers. Front Oncol (2022) 12:857743. doi: 10.3389/fonc.2022.857743 35463352 PMC9021824

[3] Zhang Q Zhong C Shen J Chen S Jia Y Duan S. Emerging role of LINC00461 in cancer. BioMed Pharmacother (2022) 152:113239. doi: 10.1016/j.biopha.2022.113239 35679722

[4] Wang KC Chang HY. Molecular mechanisms of long noncoding RNAs. Mol Cell (2011) 43(6):904–14. doi: 10.1016/j.molcel.2011.08.018 PMC3199020 21925379

[5] Lau E. Non-coding RNA: Zooming in on lncRNA functions. Nat Rev Genet (2014) 15(9):574–5. doi: 10.1038/nrg3795 25048169

[6] Charles Richard JL Eichhorn PJA. Platforms for investigating LncRNA functions. SLAS Technol (2018) 23(6):493–506. doi: 10.1177/2472630318780639 29945466 PMC6249642

[7] Bolha L Ravnik-Glavac M Glavac D. Long noncoding RNAs as biomarkers in cancer. Dis Markers (2017) 2017:7243968. doi: 10.1155/2017/7243968 28634418 PMC5467329

[8] Zhang Q Zhong C Duan S. The tumorigenic function of LINC00858 in cancer. BioMed Pharmacother (2021) 143:112235. doi: 10.1016/j.biopha.2021.112235 34649358

[9] Chen M Zhang M Xie L Wu S Zhong Y. LINC00324 facilitates cell proliferation through competing for miR2145p in immature ovarian teratocarcinoma. Int J Mol Med (2021) 47(1):397–407. doi: 10.3892/ijmm.2020.4800 33416104

[10] Zhao W Luo J Jiao S. Comprehensive characterization of cancer subtype associated long non-coding RNAs and their clinical implications. Sci Rep (2014) 4:6591. doi: 10.1038/srep06591 25307233 PMC4194441

[11] Zhang M Lin B Liu Y Huang T Chen M Lian D. LINC00324 affects non-small cell lung cancer cell proliferation and invasion through regulation of the miR-139-5p/IGF1R axis. Mol Cell Biochem (2020) 473(1-2):193–202. doi: 10.1007/s11010-020-03819-2 32734536

[12] Chen H Wei L Luo M Wang X Zhu C Huang H. LINC00324 suppresses apoptosis and autophagy in nasopharyngeal carcinoma through upregulation of PAD4 and activation of the PI3K/AKT signaling pathway. Cell Biol Toxicol (2021). doi: 10.1007/s10565-021-09632-x 34322788

[13] Pan ZH Guo XQ Shan J Luo SX. LINC00324 exerts tumor-promoting functions in lung adenocarcinoma *via* targeting miR-615-5p/AKT1 axis. Eur Rev Med Pharmacol Sci (2018) 22(23):8333–42. doi: 10.26355/eurrev_201812_16531 30556874

[14] Wang S Cheng Y Yang P Qin G. Silencing of long noncoding RNA LINC00324 interacts with MicroRNA-3200-5p to attenuate the tumorigenesis of gastric cancer *via* regulating BCAT1. Gastroenterol Res Pract (2020) 2020:4159298. doi: 10.1155/2020/4159298 32855634 PMC7442994

[15] Zou Z Ma T He X Zhou J Ma H Xie M. Long intergenic non-coding RNA 00324 promotes gastric cancer cell proliferation *via* binding with HuR and stabilizing FAM83B expression. Cell Death Dis (2018) 9(7):717. doi: 10.1038/s41419-018-0758-8 29915327 PMC6006375

[16] Wu S Gu Z Wu Y Wu W Mao B Zhao S. LINC00324 accelerates the proliferation and migration of osteosarcoma through regulating WDR66. J Cell Physiol (2020) 235(1):339–48. doi: 10.1002/jcp.28973 31225659

[17] Gao J Dai C Yu X Yin XB Zhou F. Long noncoding RNA LINC00324 exerts protumorigenic effects on liver cancer stem cells by upregulating fas ligand *via* PU box binding protein. FASEB J (2020) 34(4):5800–17. doi: 10.1096/fj.201902705RR 32128906

[18] Ni X Xie JK Wang H Song HR. Knockdown of long non-coding RNA LINC00324 inhibits proliferation, migration and invasion of colorectal cancer cell *via* targeting miR-214-3p. Eur Rev Med Pharmacol Sci (2019) 23(24):10740–50. doi: 10.26355/eurrev_201912_19775 31858541

[19] Dong Y Wan G Yan P Qian C Li F Peng G. Long noncoding RNA LINC00324 promotes retinoblastoma progression by acting as a competing endogenous RNA for microRNA-769-5p, thereby increasing STAT3 expression. Aging (Albany NY) (2020) 12(9):7729–46. doi: 10.18632/aging.103075 PMC7244063 32369777

[20] Wan JF Wan JY Dong C Li L. Linc00324 promotes the progression of papillary thyroid cancer *via* regulating notch signaling pathway. Eur Rev Med Pharmacol Sci (2020) 24(12):6818–24. doi: 10.26355/eurrev_202006_21671 32633374

[21] Chen Y Wu Y Chen R Xu C Chen Q. LncRNA LINC00324 is upregulated in intervertebral disk degeneration and upregulates FasL in nucleus pulposus cells. Mol Cell Biochem (2021) 476(5):1995–2000. doi: 10.1007/s11010-021-04058-9 33511550

[22] Wang B Zhang Y Zhang H Lin F Tan Q Qin Q. Long intergenic non-protein coding RNA 324 prevents breast cancer progression by modulating miR-10b-5p. Aging (Albany NY) (2020) 12(8):6680–99. doi: 10.18632/aging.103021 PMC7202516 32305959

[23] Xu J Li Z Su Q Zhao J Ma J. Suppression of long noncoding RNA LINC00324 restricts cell proliferation and invasion of papillary thyroid carcinoma through downregulation of TRIM29 *via* upregulating microRNA-195-5p. Aging (Albany NY) (2020) 12(24):26000–11. doi: 10.18632/aging.202219 PMC7803523 33318312

[24] Pereira B Billaud M Almeida R. RNA-Binding proteins in cancer: Old players and new actors. Trends Cancer (2017) 3(7):506–28. doi: 10.1016/j.trecan.2017.05.003 28718405

[25] Zhang Y Yu S Zhang Z Zhao G Xu J. Long non-coding RNA AK096174 promotes cell proliferation and invasion in gastric cancer by regulating WDR66 expression. Biosci Rep (2018) 38(4):3154–65. doi: 10.1042/BSR20180277 PMC6050193 29717028

[26] Shen CQ Yan TT Liu W Zhu XQ Tian XL Fu XL. High expression of FAM83B predicts poor prognosis in patients with pancreatic ductal adenocarcinoma and correlates with cell cycle and cell proliferation. J Cancer (2017) 8(16):3154–65. doi: 10.7150/jca.20086 PMC5665031 29158787

[27] Liu D Li Y Luo G Xiao X Tao D Wu X. LncRNA SPRY4-IT1 sponges miR-101-3p to promote proliferation and metastasis of bladder cancer cells through up-regulating EZH2. Cancer Lett (2017) 388:281–91. doi: 10.1016/j.canlet.2016.12.005 27998761

[28] Riedemann J Macaulay VM. IGF1R signalling and its inhibition. Endocr Relat Cancer (2006) 13 Suppl 1:S33–43. doi: 10.1677/erc.1.01280 17259557

[29] Nebenfuehr S Kollmann K Sexl V. The role of CDK6 in cancer. Int J Cancer (2020) 147(11):2988–95. doi: 10.1002/ijc.33054 PMC7586846 32406095

[30] Li N Zeng J Sun F Tong X Meng G Wu C. p27 inhibits CDK6/CCND1 complex formation resulting in cell cycle arrest and inhibition of cell proliferation. Cell Cycle (2018) 17(19-20):2335–48. doi: 10.1080/15384101.2018.1526598 PMC6237435 30317923

[31] Shadfan M Lopez-Pajares V Yuan ZM. MDM2 and MDMX: Alone and together in regulation of p53. Transl Cancer Res (2012) 1(2):88–9. PMC3448287 23002429

[32] Yuzhalin AE Gordon-Weeks AN Tognoli ML Jones K Markelc B Konietzny R. Colorectal cancer liver metastatic growth depends on PAD4-driven citrullination of the extracellular matrix. Nat Commun (2018) 9(1):4783. doi: 10.1038/s41467-018-07306-7 30429478 PMC6235861

[33] Yu H Lee H Herrmann A Buettner R Jove R. Revisiting STAT3 signalling in cancer: new and unexpected biological functions. Nat Rev Cancer (2014) 14(11):736–46. doi: 10.1038/nrc3818 25342631

[34] Liu HW Bi WT Huang HT Li RX Xi Q Feng L. Satb1 promotes schwann cell viability and migration *via* activation of PI3K/AKT pathway. Eur Rev Med Pharmacol Sci (2018) 22(13):4268–77. doi: 10.26355/eurrev_201807_15423 30024617

[35] Revathidevi S Munirajan AK. Akt in cancer: Mediator and more. Semin Cancer Biol (2019) 59:80–91. doi: 10.1016/j.semcancer.2019.06.002 31173856

[36] Hsu CY Yanagi T Ujiie H. TRIM29 in cutaneous squamous cell carcinoma. Front Med (Lausanne) (2021) 8:804166. doi: 10.3389/fmed.2021.804166 34988104 PMC8720877

[37] Stuelten CH Parent CA Montell DJ. Cell motility in cancer invasion and metastasis: insights from simple model organisms. Nat Rev Cancer (2018) 18(5):296–312. doi: 10.1038/nrc.2018.15 29546880 PMC6790333

[38] Lamouille S Xu J Derynck R. Molecular mechanisms of epithelial-mesenchymal transition. Nat Rev Mol Cell Biol (2014) 15(3):178–96. doi: 10.1038/nrm3758 PMC4240281 24556840

[39] Aiello NM Kang Y. Context-dependent EMT programs in cancer metastasis. J Exp Med (2019) 216(5):1016–26. doi: 10.1084/jem.20181827 PMC6504222 30975895

[40] Wang L Wang E Prado Balcazar J Wu Z Xiang K Wang Y. Chromatin remodeling of colorectal cancer liver metastasis is mediated by an HGF-PU.1-DPP4 axis. Adv Sci (Weinh) (2021) 8(19):e2004673. doi: 10.1002/advs.202004673 34378358 PMC8498885

[41] Roskams T Libbrecht L Van Damme B Desmet V. Fas and fas ligand: strong co-expression in human hepatocytes surrounding hepatocellular carcinoma; can cancer induce suicide in peritumoural cells? J Pathol (2000) 191(2):150–3. doi: 10.1002/(SICI)1096-9896(200006)191:2<150::AID-PATH612>3.0.CO;2-I 10861574

[42] Fukuzawa Y Takahashi K Furuta K Tagaya T Ishikawa T Wada K. Expression of fas/fas ligand (fasL) and its involvement in infiltrating lymphocytes in hepatocellular carcinoma (HCC). J Gastroenterol (2001) 36(10):681–8. doi: 10.1007/s005350170031 11686478

[43] Fattahi S Amjadi-Moheb F Tabaripour R Ashrafi GH Akhavan-Niaki H. PI3K/AKT/mTOR signaling in gastric cancer: Epigenetics and beyond. Life Sci (2020) 262:118513. doi: 10.1016/j.lfs.2020.118513 33011222

[44] Liu X Arfman T Wichapong K Reutelingsperger CPM Voorberg J Nicolaes GAF. PAD4 takes charge during neutrophil activation: Impact of PAD4 mediated NET formation on immune-mediated disease. J Thromb Haemost (2021) 19(7):1607–17. doi: 10.1111/jth.15313 PMC8360066 33773016

[45] Jozefiak A Larska M Pomorska-Mol M Ruszkowski JJ. The IGF-1 signaling pathway in viral infections. Viruses (2021) 13(8):1488. doi: 10.3390/v13081488 34452353 PMC8402757

[46] Sun Y Liu Y Ma X Hu H. The influence of cell cycle regulation on chemotherapy. Int J Mol Sci (2021) 22(13):692359. doi: 10.3390/ijms22136923 PMC8267727 34203270

[47] Kent LN Leone G. The broken cycle: E2F dysfunction in cancer. Nat Rev Cancer (2019) 19(6):326–38. doi: 10.1038/s41568-019-0143-7 31053804

[48] Hamilton E Infante JR. Targeting CDK4/6 in patients with cancer. Cancer Treat Rev (2016) 45:129–38. doi: 10.1016/j.ctrv.2016.03.002 27017286

[49] Curylova L Ramos H Saraiva L Skoda J. Noncanonical roles of p53 in cancer stemness and their implications in sarcomas. Cancer Lett (2022) 525:131–45. doi: 10.1016/j.canlet.2021.10.037 34742870

[50] Shamloo B Usluer S. p21 in cancer research. Cancers (Basel) (2019) 11(8):1178. doi: 10.3390/cancers11081178 31416295 PMC6721478

[51] Mansilla SF de la Vega MB Calzetta NL Siri SO Gottifredi V. CDK-independent and PCNA-dependent functions of p21 in DNA replication. Genes (Basel) (2020) 11(6):593. doi: 10.3390/genes11060593 32481484 PMC7349641

[52] Karimian A Ahmadi Y Yousefi B. Multiple functions of p21 in cell cycle, apoptosis and transcriptional regulation after DNA damage. DNA Repair (Amst) (2016) 42:63–71. doi: 10.1016/j.dnarep.2016.04.008 27156098

[53] Locatelli M Curigliano G. Notch inhibitors and their role in the treatment of triple negative breast cancer: promises and failures. Curr Opin Oncol (2017) 29(6):411–27. doi: 10.1097/CCO.0000000000000406 28914645

[54] Meurette O. Shaping of the tumor microenvironment by notch signaling. Adv Exp Med Biol (2020) 1223:1–16. doi: 10.1007/978-3-030-35582-1_1 32030682

[55] Jin W. Role of JAK/STAT3 signaling in the regulation of metastasis, the transition of cancer stem cells, and chemoresistance of cancer by epithelial-mesenchymal transition. Cells (2020) 9(1):217. doi: 10.3390/cells9010217 31952344 PMC7017057

[56] Johnson DE O'Keefe RA Grandis JR. Targeting the IL-6/JAK/STAT3 signalling axis in cancer. Nat Rev Clin Oncol (2018) 15(4):234–48. doi: 10.1038/nrclinonc.2018.8 PMC5858971 29405201

[57] Huynh J Etemadi N Hollande F Ernst M Buchert M. The JAK/STAT3 axis: A comprehensive drug target for solid malignancies. Semin Cancer Biol (2017) 45:13–22. doi: 10.1016/j.semcancer.2017.06.001 28647610

[58] Li H An X Li Q Yu H Li Z. Construction and analysis of competing endogenous RNA network of MCF-7 breast cancer cells based on the inhibitory effect of 6-thioguanine on cell proliferation. Oncol Lett (2021) 21(2):104. doi: 10.3892/ol.2020.12365 33376537 PMC7751352

[59] Hartung M Anastasi E Mamdouh ZM Nogales C Schmidt H Baumbach J. Cancer driver drug interaction explorer. Nucleic Acids Res (2022) 50(W1):W138–44. doi: 10.1093/nar/gkac384 PMC9252786 35580047

[60] Ghafouri-Fard S Safarzadeh A Hussen BM Taheri M Rashnoo F. A concise review on the role of LINC00324 in different cancers. Pathol Res Pract (2022) 240:154192. doi: 10.1016/j.prp.2022.154192 36399929

[61] Kuo WH Chang YY Lai LC Tsai MH Hsiao CK Chang KJ. Molecular characteristics and metastasis predictor genes of triple-negative breast cancer: a clinical study of triple-negative breast carcinomas. PloS One (2012) 7(9):e45831. doi: 10.1371/journal.pone.0045831 23049873 PMC3458056

[62] Statello L Guo CJ Chen LL Huarte M. Gene regulation by long non-coding RNAs and its biological functions. Nat Rev Mol Cell Biol (2021) 22(2):96–118. doi: 10.1038/s41580-020-00315-9 33353982 PMC7754182

[63] Choi J Kang M Nam SH Lee GH Kim HJ Ryu J. Bidirectional signaling between TM4SF5 and IGF1R promotes resistance to EGFR kinase inhibitors. Lung Cancer (2015) 90(1):22–31. doi: 10.1016/j.lungcan.2015.06.023 26190015

[64] Sen T Sen N Brait M Begum S Chatterjee A Hoque MO. DeltaNp63alpha confers tumor cell resistance to cisplatin through the AKT1 transcriptional regulation. Cancer Res (2011) 71(3):1167–76. doi: 10.1158/0008-5472.CAN-10-1481 PMC3076926 21266360

[65] Tu B Zhu J Liu S Wang L Fan Q Hao Y. Mesenchymal stem cells promote osteosarcoma cell survival and drug resistance through activation of STAT3. Oncotarget (2016) 7(30):48296–308. doi: 10.18632/oncotarget.10219 PMC5217018 27340780

