# Supplementary material for: LINC00324 in cancer: Regulatory and therapeutic implications
Source: Front Oncol. 2022 Dec 22;12:1039366. doi: 10.3389/fonc.2022.1039366 (PMC9815511; doi:10.3389/fonc.2022.1039366)
Supplement: Supplementary file 1 [file DataSheet_1.docx]

**Text**

**1. Abnormal expression of LINC00324 in tumors**

We downloaded the TPM expression data of LINC00324 in TCGA, TARGET, and GTEx for 32 cancer types from the UCSC database (https://xenabrowser.net/ ) and performed log2(TPM+1) transformation. We calculated the quantile rank of LINC00324 among all non-zero-expressed lncRNAs in each of these 32 cancer types. As shown in Figure S1A, LINC00324 was highly expressed (0.75-1.0 quantile, Q4) in 32 tumors. We compared the difference in expression of LINC00324 between normal and tumor samples in each cancer type (R version 4.1.1, unpaired Wilcoxon test). As shown in Figure S1B, significant upregulation of LINC00324 was observed in 13 TCGA tumors, including COAD, ESCA, GBM, KURC, LGG, LIHC, LUAD, PAAD, PRAD, SARC, STAD, TGCT and THCA. LINC00324 was significantly downregulated in 11 tumors, including ACC, AML, ALL, KICH, NBL, OV, PCPG, READ, SKCM, UCEC, and UCS. No significant differences were found for LINC00324 in the other 8 cancers, including BLCA, BRCA, CESC, CHOL, HNSC, KIRP, LUSC, and THYM.

As shown in Table S1, the results of seven TCGA tumors were consistent with previous reports, including COAD, LGG, LIHC, LUAD, SARC, STAD, and THCA. However, the results in TCGA-BRCA and HNSC differ from existing results. Existing studies showed that LINC00324 was down-regulated and up-regulated in BC (1)and NPC(2), while in TCGA-BRCA and TCGA-HNSC, LINC00324 expression was not significantly different between cancerous and non-cancerous tissues(Table S1). This may be due to differences in cancer subtypes or detection methods, or samples in the TCGA database. Additionally, IOTs were not included in the TCGA database and were therefore not analyzed in the bioinformatics analysis.

We downloaded expression data (TPM) of LINC00324 in TCGA, TARGET for 32 cancer types from https://xenabrowser.net/ UCSC database and performed log2(TPM+1) transformation. The expression correlation of LINC00324 with other genes was calculated using the R package "psych". These genes include RNA modification-related genes (m1A(3), m5C(4), and m6A(5)) and adjacent genes of LINC00324 (CTC1 and AURKB).

As shown in Figure S2A, in AML, LINC00324 was positively correlated with the expression of various RNA-modifying genes. In ALL, however, LINC00324 was not associated with most RNA-modifying genes. Previous analysis found that LINC00324 was down-regulated in AML. Whether LINC00324 down-regulation in AML is associated with RNA modification requires further investigation. In GBM, LINC00324 expression was significantly negatively correlated with some RNA-modifying gene expression. Previous studies have found that LINC00324 is significantly up-regulated in GBM, and it is necessary to investigate whether the up-regulation of LINC0032 in GBM is related to RNA modification in the future. In renal and reproductive system-related cancers, LINC00324 expression was significantly positively correlated with RNA modifications. Furthermore, in digestive system-related cancers (including CHOL, COAD, ESCA, and READ), LINC00324 expression was not significantly associated with most RNA-modifying genes.

Notably, LRPPRC is not only an m6A-modified reader but also inhibits mitophagy (6). In 12 cancers, LINC00324 expression was significantly negatively correlated with LRPPRC expression. The adjacent genes of LINC00324 include the upstream gene CTC1 with a distance of 3783 bp and the downstream gene AURKB with a distance of 10042 bp (Fig. S2B). CTC1 can inhibit telomere shortening and apoptosis (7). AURKB can promote cell mitosis (8). As shown in Figure S2A, in 23 cancers, the expression of LINC00324 was significantly positively correlated with the expression of the adjacent gene CTC1. LINC00324 was significantly positively correlated with AURKB expression in 5 cancers and negatively correlated in 3 cancers, suggesting that the interaction of LINC00324 with AURKB is tumor-specific.

**Table S1. Comparison of TCGA analysis and reported results**

| **cancer** | **Sample size**  **(T/N)** | **LINC00324 expression in TCGA** | **LINC00324 expression in the reported studies** |
| --- | --- | --- | --- |
| ACC | 77/121 | Down-regulated | Not studied |
| ALL | 194/337 | Down-regulated | Not studied |
| AML | 367/337 | Down-regulated | Not studied |
| BLCA | 407/28 | ns | Not studied |
| BRCA | 1092/292 | ns | Down-regulated in BC tissues and BC cells (MDA-MB-231 and MCF-7 ) (1) |
| CESC | 306/13 | ns | Not studied |
| CHOL | 36/9 | ns | Not studied |
| COAD | 290/345 | Up-regulated | Up-regulated in CRC cells (SW620, HCT15, SW480, and HCT116) (9) |
| ESCA | 182/664 | Up-regulated | Not studied |
| GBM | 166/1149 | Up-regulated | Not studied |
| HNSC | 520/44 | ns | Up-regulated in NPC tissues and cells(5-8F, 6-10B, and NP69) (2) |
| KICH | 66/52 | Down-regulated | Not studied |
| KIRC | 544/99 | Up-regulated | Not studied |
| KIRP | 289/59 | ns | Not studied |
| LGG | 523/1144 | Up-regulated | Not studied |
| LIHC | 371/160 | Up-regulated | Up-regulated in HCC tissues and HCC cells (HuH-7) (10) |
| LUAD | 515/346 | Up-regulated | Up-regulated in LUAD tissues and LUAD cells (A549, PC-9, H1650, and SPCA1) (11) |
| LUSC | 498/337 | ns | Not studied |
| NBL | 162/274 | Down-regulated | Up-regulated in RB cells(Y79 and WERI-RB-1)(12) |
| OV | 426/88 | Down-regulated | Not studied |
| PAAD | 179/169 | Up-regulated | Not studied |
| PCPG | 182/130 | Down-regulated | Not studied |
| PRAD | 496/152 | Up-regulated | Not studied |
| READ | 93/10 | Down-regulated | Not studied |
| SARC | 262/398 | Up-regulated | Up-regulated in SaOS cells(hFOB1.19, 143B, MG-63, Saos-2 and HOS)(13) |
| SKCM | 469/558 | Down-regulated | Not studied |
| STAD | 303/26 | Up-regulated | Up-regulated in STAD tissues and STAD cells (AGS, MGC803, MKN-45,  SGC7901, and BGC823) (14) |
| THCA | 512/337 | Up-regulated | Up-regulated in THCA tissues and THCA cells (B-CPAP, KTC-1, TPC1, and K1) (15) |
| THYM | 119/2 | ns | Not studied |
| UCEC | 181/101 | Down-regulated | Not studied |
| UCS | 57/78 | Down-regulated | Not studied |

ns, not significant; ACC, Adrenocortical carcinoma; ALL, Acute lymphoblastic leukemia; AML, Acute myeloid leukemia; BLCA, Bladder urothelial carcinoma; BRCA, Breast invasive carcinoma; CESC, Cervical squamous cell carcinoma and endocervical adenocarcinoma; CHOL, Cholangiocarcinoma; COAD, Colon adenocarcinoma; ESCA, Esophageal carcinoma; GBM, Glioblastoma multiforme; HNSC, Head and Neck squamous cell carcinoma; KICH, Kidney chromophobe; KIRC, Kidney renal clear cell carcinoma; KIRP, Kidney renal papillary cell carcinoma; LGG, Brain lower grade glioma; LIHC, Liver hepatocellular carcinoma; LUAD, Lung adenocarcinoma; LUSC, Lung squamous cell carcinoma; NBL, Neuroblastoma; OV, Ovarian serous cystadenocarcinoma; PAAD, Pancreatic adenocarcinoma; PCPG, Pheochromocytoma and Paraganglioma; PRAD, Prostate adenocarcinoma; READ, Rectum adenocarcinoma; SARC, Sarcoma; STAD, Stomach adenocarcinoma; SKCM, Skin cutaneous melanoma; TGCT, Testicular germ cell tumors; THCA, Thyroid carcinoma; THYM, Thymoma; UCEC, Uterine corpus endometrial carcinoma; UCS, Uterine carcinosarcoma

**Figure S1. A pan-cancer analysis of LINC00324**

**
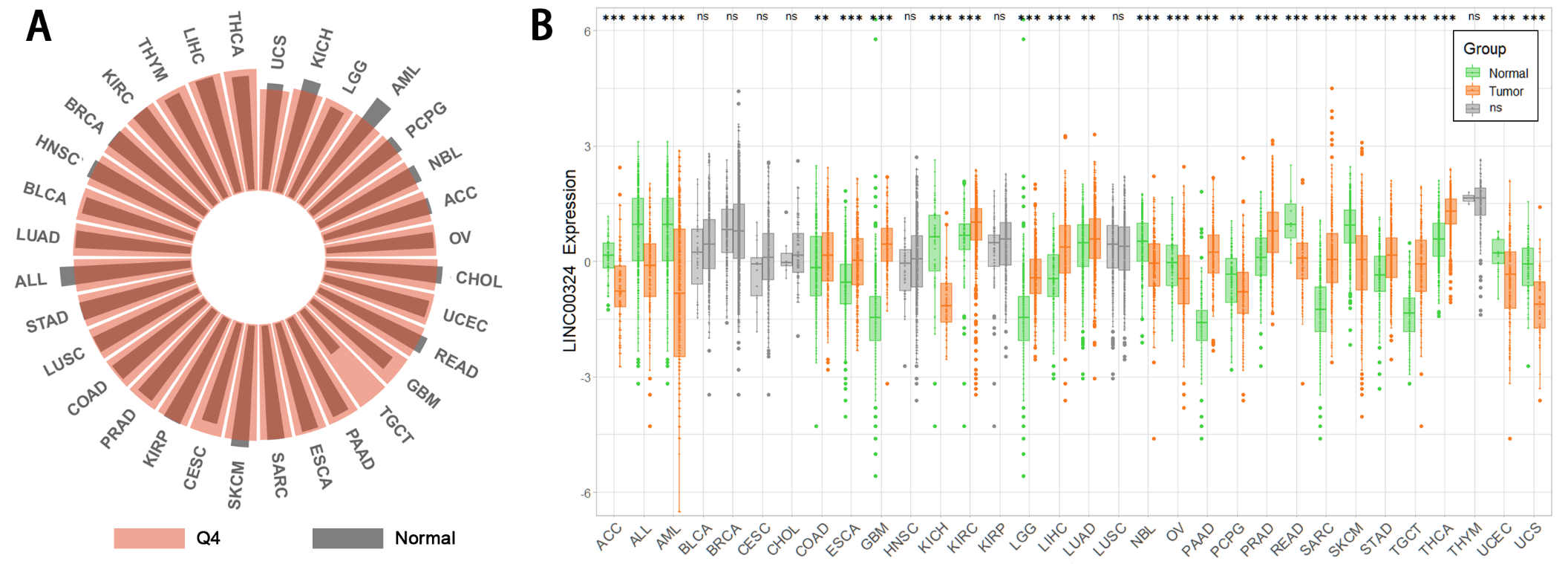
**

1. quantile expression of LINC00324 in 32 cancer types; (B) LINC00324 is dysregulated in 32 cancer types. (*** means p < 0.001, ** means p<0.01, * means p<0.05, ns means no significant difference). ACC, Adrenocortical carcinoma; ALL, Acute lymphoblastic leukemia; AML, Acute myeloid leukemia; BLCA, Bladder urothelial carcinoma; BRCA, Breast invasive carcinoma; CESC, Cervical squamous cell carcinoma and endocervical adenocarcinoma; CHOL, Cholangiocarcinoma; COAD, Colon adenocarcinoma; ESCA, Esophageal carcinoma; GBM, Glioblastoma multiforme; HNSC, Head and Neck squamous cell carcinoma; KICH, Kidney chromophobe; KIRC, Kidney renal clear cell carcinoma; KIRP, Kidney renal papillary cell carcinoma; LGG, Brain lower grade glioma; LIHC, Liver hepatocellular carcinoma; LUAD, Lung adenocarcinoma; LUSC, Lung squamous cell carcinoma; NBL, Neuroblastoma; OV, Ovarian serous cystadenocarcinoma; PAAD, Pancreatic adenocarcinoma; PCPG, Pheochromocytoma and Paraganglioma; PRAD, Prostate adenocarcinoma; READ, Rectum adenocarcinoma; SARC, Sarcoma; STAD, Stomach adenocarcinoma; SKCM, Skin cutaneous melanoma; TGCT, Testicular germ cell tumors; THCA, Thyroid carcinoma; THYM, Thymoma; UCEC, Uterine corpus endometrial carcinoma; UCS, Uterine carcinosarcoma.

**Figure S2. Correlation analysis of LINC00324 with RNA-modifying genes and adjacent genes**

**
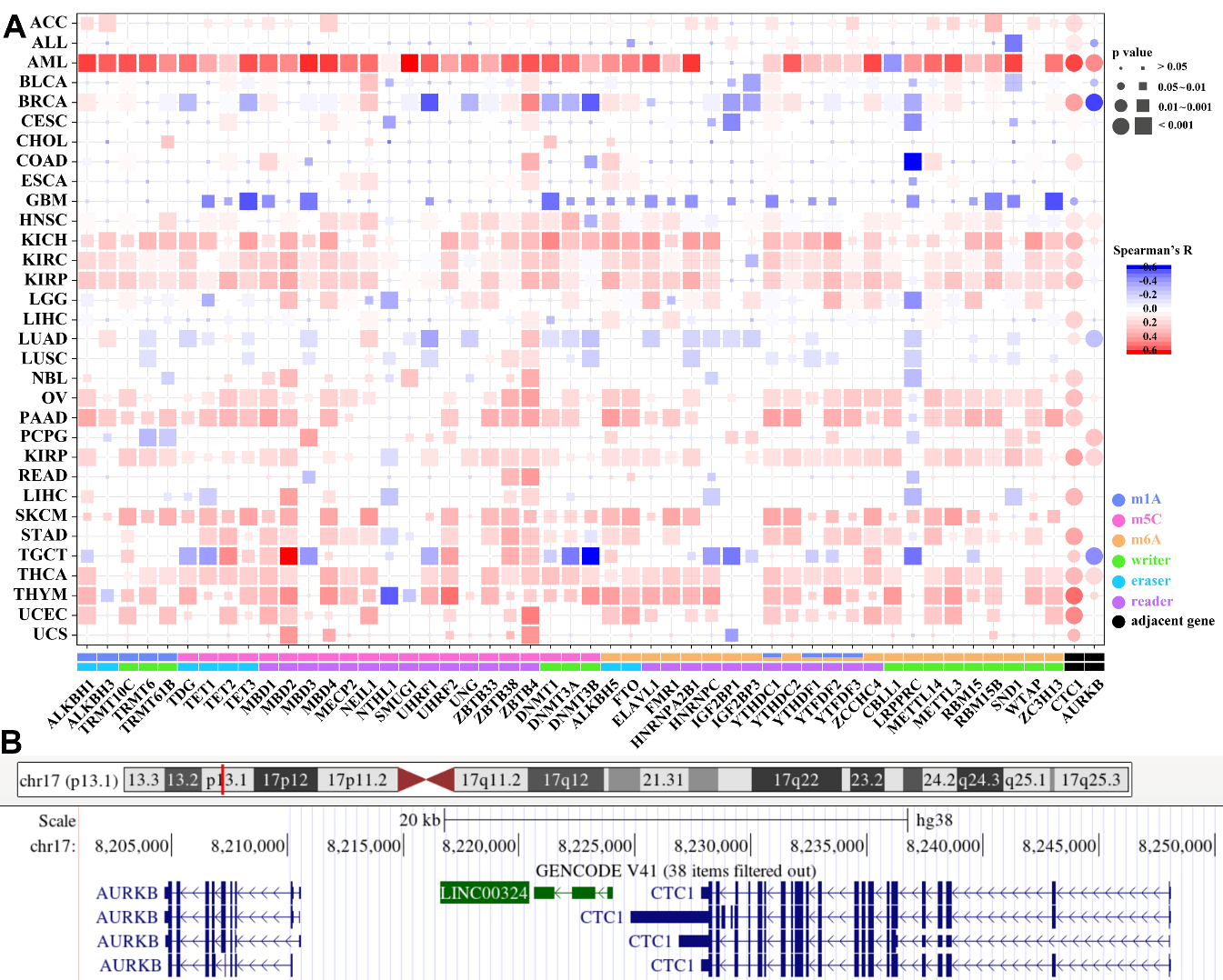
**

(A) The relationship between LINC00324 and RNA-modifying genes and adjacent genes (B) The locations of LINC00324 and its adjacent genes. ACC, Adrenocortical carcinoma; ALL, Acute lymphoblastic leukemia; AML, Acute myeloid leukemia; BLCA, Bladder urothelial carcinoma; BRCA, Breast invasive carcinoma; CESC, Cervical squamous cell carcinoma and endocervical adenocarcinoma; CHOL, cholangiocarcinoma; COAD, Colon adenocarcinoma; ESCA, Esophageal carcinoma; GBM, Glioblastoma multiforme; HNSC, Head and Neck squamous cell carcinoma; KICH, Kidney chromophobe; KIRC, Kidney renal clear cell carcinoma; KIRP, Kidney renal papillary cell carcinoma; LGG, Brain lower grade glioma; LIHC, Liver hepatocellular carcinoma; LUAD, Lung adenocarcinoma; LUSC, Lung squamous cell carcinoma; NBL, Neuroblastoma; OV, Ovarian serous cystadenocarcinoma; PAAD, Pancreatic adenocarcinoma; PCPG, Pheochromocytoma and Paraganglioma; PRAD, Prostate adenocarcinoma; READ, Rectum adenocarcinoma; SARC, Sarcoma; STAD, Stomach adenocarcinoma; SKCM, Skin cutaneous melanoma; TGCT, Testicular germ cell tumors; THCA, Thyroid carcinoma; THYM, Thymoma; UCEC, Uterine corpus endometrial carcinoma; UCS, Uterine carcinosarcoma.

**Reference**

1. Wang B, Zhang Y, Zhang H, Lin F, Tan Q, Qin Q, et al. Long intergenic non-protein coding RNA 324 prevents breast cancer progression by modulating miR-10b-5p. *Aging (Albany NY)* (2020) 12(8):6680-99. Epub 2020/04/20. doi: 10.18632/aging.103021. PubMed PMID: 32305959; PubMed Central PMCID: PMCPMC7202516.

2. Chen H, Wei L, Luo M, Wang X, Zhu C, Huang H, et al. LINC00324 suppresses apoptosis and autophagy in nasopharyngeal carcinoma through upregulation of PAD4 and activation of the PI3K/AKT signaling pathway. *Cell Biol Toxicol* (2021). Epub 2021/07/30. doi: 10.1007/s10565-021-09632-x. PubMed PMID: 34322788.

3. Zhao M, Shen S, Xue C. A Novel m1A-Score Model Correlated With the Immune Microenvironment Predicts Prognosis in Hepatocellular Carcinoma. *Front Immunol* (2022) 13:805967. Epub 2022/04/12. doi: 10.3389/fimmu.2022.805967. PubMed PMID: 35401564; PubMed Central PMCID: PMCPMC8987777.

4. Hu J, Othmane B, Yu A, Li H, Cai Z, Chen X, et al. 5mC regulator-mediated molecular subtypes depict the hallmarks of the tumor microenvironment and guide precision medicine in bladder cancer. *BMC Med* (2021) 19(1):289. Epub 2021/11/28. doi: 10.1186/s12916-021-02163-6. PubMed PMID: 34836536; PubMed Central PMCID: PMCPMC8627095.

5. Zhao Q, Zhao Y, Hu W, Zhang Y, Wu X, Lu J, et al. m(6)A RNA modification modulates PI3K/Akt/mTOR signal pathway in Gastrointestinal Cancer. *Theranostics* (2020) 10(21):9528-43. Epub 2020/08/31. doi: 10.7150/thno.42971. PubMed PMID: 32863943; PubMed Central PMCID: PMCPMC7449908.

6. Song K, Li B, Chen YY, Wang H, Liu KC, Tan W, et al. LRPPRC regulates metastasis and glycolysis by modulating autophagy and the ROS/HIF1-alpha pathway in retinoblastoma. *Mol Ther Oncolytics* (2021) 22:582-91. Epub 2021/10/01. doi: 10.1016/j.omto.2021.06.009. PubMed PMID: 34589577; PubMed Central PMCID: PMCPMC8450181.

7. Luo YM, Xia NX, Yang L, Li Z, Yang H, Yu HJ, et al. CTC1 increases the radioresistance of human melanoma cells by inhibiting telomere shortening and apoptosis. *Int J Mol Med* (2014) 33(6):1484-90. Epub 2014/04/11. doi: 10.3892/ijmm.2014.1721. PubMed PMID: 24718655; PubMed Central PMCID: PMCPMC4055431.

8. Nie M, Wang Y, Yu Z, Li X, Deng Y, Wang Y, et al. AURKB promotes gastric cancer progression via activation of CCND1 expression. *Aging (Albany NY)* (2020) 12(2):1304-21. Epub 2020/01/27. doi: 10.18632/aging.102684. PubMed PMID: 31982864; PubMed Central PMCID: PMCPMC7053608.

9. Ni X, Xie JK, Wang H, Song HR. Knockdown of long non-coding RNA LINC00324 inhibits proliferation, migration and invasion of colorectal cancer cell via targeting miR-214-3p. *Eur Rev Med Pharmacol Sci* (2019) 23(24):10740-50. Epub 2019/12/21. doi: 10.26355/eurrev_201912_19775. PubMed PMID: 31858541.

10. Gao J, Dai C, Yu X, Yin XB, Zhou F. Long noncoding RNA LINC00324 exerts protumorigenic effects on liver cancer stem cells by upregulating fas ligand via PU box binding protein. *FASEB J* (2020) 34(4):5800-17. Epub 2020/03/05. doi: 10.1096/fj.201902705RR. PubMed PMID: 32128906.

11. Pan ZH, Guo XQ, Shan J, Luo SX. LINC00324 exerts tumor-promoting functions in lung adenocarcinoma via targeting miR-615-5p/AKT1 axis. *Eur Rev Med Pharmacol Sci* (2018) 22(23):8333-42. Epub 2018/12/18. doi: 10.26355/eurrev_201812_16531. PubMed PMID: 30556874.

12. Dong Y, Wan G, Yan P, Qian C, Li F, Peng G. Long noncoding RNA LINC00324 promotes retinoblastoma progression by acting as a competing endogenous RNA for microRNA-769-5p, thereby increasing STAT3 expression. *Aging (Albany NY)* (2020) 12(9):7729-46. Epub 2020/05/06. doi: 10.18632/aging.103075. PubMed PMID: 32369777; PubMed Central PMCID: PMCPMC7244063.

13. Wu S, Gu Z, Wu Y, Wu W, Mao B, Zhao S. LINC00324 accelerates the proliferation and migration of osteosarcoma through regulating WDR66. *J Cell Physiol* (2020) 235(1):339-48. Epub 2019/06/22. doi: 10.1002/jcp.28973. PubMed PMID: 31225659.

14. Wang S, Cheng Y, Yang P, Qin G. Silencing of Long Noncoding RNA LINC00324 Interacts with MicroRNA-3200-5p to Attenuate the Tumorigenesis of Gastric Cancer via Regulating BCAT1. *Gastroenterol Res Pract* (2020) 2020:4159298. Epub 2020/08/29. doi: 10.1155/2020/4159298. PubMed PMID: 32855634; PubMed Central PMCID: PMCPMC7442994.

15. Xu J, Li Z, Su Q, Zhao J, Ma J. Suppression of long noncoding RNA LINC00324 restricts cell proliferation and invasion of papillary thyroid carcinoma through downregulation of TRIM29 via upregulating microRNA-195-5p. *Aging (Albany NY)* (2020) 12(24):26000-11. Epub 2020/12/16. doi: 10.18632/aging.202219. PubMed PMID: 33318312; PubMed Central PMCID: PMCPMC7803523.
